# Comparison of two *Phaeodactylum tricornutum* ecotypes under nitrogen starvation and resupply reveals distinct lipid accumulation strategies but a common degradation process

**DOI:** 10.3389/fpls.2023.1257500

**Published:** 2023-09-22

**Authors:** Victor Murison, Josiane Hérault, Martine Côme, Sabrina Guinio, Alexis Lebon, Christophe Chamot, Magalie Bénard, Ludovic Galas, Benoît Schoefs, Justine Marchand, Muriel Bardor, Lionel Ulmann

**Affiliations:** ^1^ Biology of Organisms, Stress, Health and Environment, IUT Département Génie Biologique, Le Mans Université, IUML-FR 3473 CNRS, Laval, France; ^2^ Université de Rouen Normandie, INSERM, CNRS, HeRacLeS US51 UAR2026, PRIMACEN, Rouen, France; ^3^ Biology of Organisms, Stress, Health and Environment, UFR Sciences et Techniques, Le Mans Université, IUML-FR 3473 CNRS, Le Mans, France; ^4^ Université de Rouen Normandie, Laboratoire GlycoMEV UR4358, SFR Normandie Végétal FED 4277, Innovation Chimie Carnot, Rouen, France

**Keywords:** diatom, *Phaeodactylum*, lipid droplet, lipid catabolism, chloroplast, stress, nitrogen

## Abstract

**Introduction:**

*Phaeodactylum tricornutum* is a model species frequently used to study lipid metabolism in diatoms. When exposed to a nutrient limitation or starvation, diatoms are known to accumulate neutral lipids in cytoplasmic lipid droplets (LDs). Those lipids are produced partly de novo and partly from the recycle of plastid membrane lipids. Under a nitrogen resupply, the accumulated lipids are catabolized, a phenomenon about which only a few data are available. Various strains of *P. tricornutum* have been isolated around the world that may differ in lipid accumulation patterns.

**Methods:**

To get further information on this topic, two genetically distant ecotypes of *P. tricornutum* (Pt1 and Pt4) have been cultivated under nitrogen deprivation during 11 days followed by a resupply period of 3 days. The importance of cytoplasmic LDs relative to the plastid was assessed by a combination of confocal laser scanning microscopy and cell volume estimation using bright field microscopy pictures.

**Results and discussion:**

We observed that in addition to a basal population of small LDs (0.005 μm^3^ to 0.7 μm^3^) present in both strains all along the experiment, Pt4 cells immediately produced two large LDs (up to 12 μm^3^ after 11 days) while Pt1 cells progressively produced a higher number of smaller LDs (up to 7 μm^3^ after 11 days). In this work we showed that, in addition to intracellular available space, lipid accumulation may be limited by the pre-starvation size of the plastid as a source of membrane lipids to be recycled. After resupplying nitrogen and for both ecotypes, a fragmentation of the largest LDs was observed as well as a possible migration of LDs to the vacuoles that would suggest an autophagic degradation. Altogether, our results deepen the understanding of LDs dynamics and open research avenues for a better knowledge of lipid degradation in diatoms.

## 1 Introduction

Microalgae are photosynthetic microorganisms contributing to around 50% of global primary production as the main aquatic ecosystems producers (Benoiston et al., 2017). Under the same word, microalgae include a high diversity of genetically and physiologically distant phenotypic groups (Burki et al., 2020). One major phylum is the Stramenopiles, a vast group that is believed to have arisen through secondary endosymbiosis, *i.e.* after a red alga was engulfed and conserved as a plastid by an heterotroph cell (Grosche et al., 2014). This peculiar evolutionary origin resulted in a specific intracellular organization and a set of original metabolic pathways in these organisms, including diatoms (*Bacillariophyceae*). From the organization point of view, the most striking originality concerns the chloroplast that is delineated by four membranes, the outermost being continuous with the endoplasmic reticulum (ER) (Flori et al., 2016). From the metabolic point of view, diatoms possess a mixture of animal- and plant-like metabolisms such as the urea cycle and the fatty acid synthesis in the plastid (Allen et al., 2011; Zulu et al., 2018; Butler et al., 2020).

Diatom lipid metabolism raised a lot of research interest because some oleaginous species can accumulate up to 69% of their biomass as lipids (Sayanova et al., 2017), making them a possible feedstock for the production of biofuels (Mourya et al., 2022). Indeed, many diatom species possess a highly flexible carbon metabolism as an adaptation to environment variations. When exposed to a variety of stresses, including nutrient starvation (Abida et al., 2015; Alipanah et al., 2015; Alipanah et al., 2018; Heydarizadeh et al., 2019; Huang et al., 2019; Scarsini et al., 2021; Scarsini et al., 2022), changes in light quality (Sharma et al., 2020; Duarte et al., 2021), and the presence of reactive oxygen and nitrogen species (Burch and Franz, 2016; Dolch et al., 2017), diatoms reorient the carbon metabolism toward the production of energy-rich neutral lipids. These molecules are packed inside specialized and dynamic storage organelles, the lipid droplets (LDs) (Leyland et al., 2020a).

The accumulation of lipids in diatoms has been well characterized. It occurs by two main routes. First, fatty acids can be synthesized *de novo* in the plastid and then incorporated in triacylglycerol (TAG) in the ER. Secondly, the fatty acid chains of membrane lipids (including plastidial lipids) can be recycled by incorporation in TAG in the ER. Then, LDs would bud from this organelle. While the enzymes involved in these processes have been identified and partially characterized (Zulu et al., 2018), those associated with transport mechanisms as well as lipid degradation pathways remain to be described. By homology to other eukaryotes, it is hypothesized that lipids are degraded by two processes, lipolysis and lipophagy (Kong et al., 2018). Both processes imply the action of lipases to hydrolyze TAG into fatty acids and glycerol. In lipolysis, the enzymes get a direct access to lipids from the surface of LDs. By contrast, in lipophagy, whole fragments of LDs are digested in the vacuole by autophagy (Kong et al., 2018; Zienkiewicz and Zienkiewicz, 2020). Currently, only a few lipases have been characterized in diatoms (Trentacoste et al., 2013; Barka et al., 2016; Li et al., 2018; Shu et al., 2023) and many more remain to be studied, including some possibly localized in the vacuoles (Murison et al., 2023).

Hence, the lipid metabolism in diatoms is tightly linked to their complex intracellular organization. A few studies have focused on how LDs are formed during lipid accumulation by examining the number and volumes of LDs marked with a fluorescent probe in the model diatom *Phaeodactylum tricornutum* under a nitrogen (N) starvation (Wong and Franz, 2013; Jaussaud et al., 2020; Leyland et al., 2020b; Scarsini et al., 2021). The two studies conducted on the Pt4 ecotype revealed a stable number of LDs during the starvation (Wong and Franz, 2013; Leyland et al., 2020b). In contrast, in Pt1 ecotype LDs accumulated in successive subpopulations, with a first subpopulation appearing in the first days of starvation and growing to a maximum volume, before the formation of two other subpopulations in which LD volume progressively increase until lipid accumulation is limited by cytosolic space availability (Jaussaud et al., 2020). A first comparison of Pt1 and Pt4 LDs characteristics was carried out after 18 days of N starvation, retrieving the general features previously identified in each ecotype and highlighting that Pt4 cells are larger than Pt1 ones (Scarsini et al., 2021). However, this study did not assess the dynamics of LDs accumulation and cell volume along the starvation. Additionally, how these ecotypes consume their different LDs once nitrogen is resupplied is currently unknown.

To understand further the above-described phenomena, we investigated the strategies of LDs accumulation in *P. tricornutum* Pt1 and Pt4 ecotypes and monitored their degradation dynamics under N starvation and N resupply. Confocal laser scanning microscopy (CLSM) and bright field microscopy allowed us to decipher the lipid accumulation and degradation strategies of two genetically distant ecotypes of *P. tricornutum*. Finally, we also questioned the relationship existing between cell volume and the amplitude of lipid accumulation.

## 2 Materials and methods

### 2.1 Cultivation conditions of *Phaeodactylum tricornutum*, nitrate starvation and resupply and growth monitoring

Triplicate non agitated cultures of *P. tricornutum* ecotypes Pt1 and Pt4 (respectively CCMP 2561 and UTEX 646) were conducted. Cells were grown in 500 mL Erlenmeyer flasks containing 200 mL of autoclaved f/2 medium (Guillard and Ryther, 1962). Cultures were inoculated at a cellular density of approximately 10^5^ cells/mL. Temperature was maintained at 19°C and light intensity was provided at 55 µmol/m²/s with daylight fluorescent tubes (LuxLine Plus T8 F58W/865 G13, Sylvania Lighting) following a light-dark cycle of 16h-8h. This growth intensity was chosen because it allowed to drive similar relative electron transport rates in the different strains studied here as determined by tracing Photosynthesis-Irradiance (PI) curves using a Dual-PAM 100 (Walz, Germany) operated with the default routine (**Supplementary Data S1**).

After a 7 days-period of N replete growth in f/2 medium, cells were transferred to a f/2 nitrogen free medium (N_0_) for 11 days. For each ecotype, the content of each Erlenmeyer flask was centrifuged at 2,000×*g* for 15 min and washed twice with N_0_ medium. Triplicates were then resuspended in fresh N_0_ medium and finally pooled in one sterile bottle before being equally redistributed between three new sterile 500 mL Erlenmeyer flasks in a final volume of 200 mL of N_0_. After the 11 days period of N starvation, a N resupply experiment of 72 hours was conducted by adding in each flask 0.006 mg NaNO_3_/10^6^ cells/day, according to the upper bound of NO_3_^-^ uptake by *P. tricornutum* cells determined in Levering et al. (2016). Samples were harvested at 6 timepoints: right after the medium is changed to N_0_ (D0), after 3, 7 and 11 days of N starvation (D3, D7, D11) and after 24 and 72 hours post N resupply (h24 and h72). All samples were collected at the same hour in the morning to limit circadian influence between timepoints. To prevent carbon limitation, the medium was supplemented with 7.5 mL/L of NaHCO_3_ (8%) upon initiation of the culture and after the switch to N_0_ medium. Cell density variations were monitored by counting the cells with a Malassez hemocytometer.

### 2.2 Staining with BODIPY 505/515 and confocal imaging

Living cells of Pt1 and Pt4 were stained with BODIPY 505/515 at a final concentration of 1 µg/mL in DMSO. After staining, 10 µL of culture were sedimented during 5 min on a glass slide and covered with a glass coverslip.

Cells were observed with a Leica TCS SP8 upright confocal microscope equipped with a ×63 oil-immersion objective (Leica Microsystems, Nanterre, France). Chlorophyll and BODIPY 505/515 were excited at 488 nm, and light was collected on three channels: one photomultiplicator detector for transmitted light, and two hybrid detectors operated in “counting” mode for the fluorescence emitted by BODIPY 505/515 (em: 505-550 nm) and chlorophyll (em: 650-700 nm). Images were acquired as 3D images (xyz) with a step size of 0.1 µm between slices in the z dimension, and a zoom factor of ×2.5. In this manuscript, chlorophyll autofluorescence is displayed in magenta while BODIPY fluorescence is displayed in green.

### 2.3 Confocal image analysis and 3D modelling of plastid and lipid droplets

Raw images from confocal acquisitions were processed through deconvolution with the software Huygens professional 4.5.1 (Scientific Volume Imaging, Hilversum, Netherlands). Deconvoluted images were further processed using Fiji freeware (ImageJ) to adjust brightness and contrast of each channel and produce maximum intensity projections on the z axis.

Deconvoluted images were also used as input for 3D modelling of LDs and of the area where chlorophyll fluorescence was detected (*i.e.* thylakoids) with IMARIS 9.0 (Oxford Instruments, United Kingdom). Cells were individually selected when images contained multiple cells. Then, for each cell, 3D volumes were created for each LD and for thylakoids using values of the “smooth” and “background” parameters specific for thylakoids (respectively 0.3 µm and 0.2 µm), Pt1 LDs (respectively 0.1 µm and 0.2 µm) and Pt4 LDs (respectively 0.1 µm and 0.4 µm), so that the model matches the signal. The splitting of touching objects was also allowed. To prevent false positive modelling, the transmitted light channel was used to visualize the limits of each cell and remove objects eventually found outside the cells. Finally, the volume and the barycenter coordinates of each object were retrieved.

Various parameters were derived from the modelled LDs for each cell: the number of LDs, the sum of individual LD volumes (total LD volume), the fraction of total LD volume that is occupied by each individual LD and the Euclidian distance from each LD to the plastid of their cell calculated according to equation 1.


(Eq. 1) $${\text{Distance}}_{\text{LD−plastid}}=\;\sqrt{{\left({\text{x}}_{\text{LD}}\text{ −}{\text{x}}_{\text{P}}\right)}^{2}+{\left({\text{y}}_{\text{LD}}\text{ −}{\text{y}}_{\text{P}}\right)}^{2}+{\left({\text{z}}_{\text{LD}}\text{ −}{\text{z}}_{\text{P}}\right)}^{2}}$$

where x_LD_, y_LD_, z_LD_ and x_P_, y_P_, z_P_ are the spatial coordinates of the corresponding LD and the plastid barycenters.

### 2.4 Quantification of chlorophyll content

For pigment extraction, 2 mL of culture volume were harvested by centrifugation (14,500×*g*, 10 min, 4°C) and then submitted to mechanical lysis (TissueLyser LT, Qiagen, Les Ulis, France). Acid-washed glass beads (425-600 µm, Sigma-Aldrich, Saint-Quentin Fallavier, France) and a volume of 500 µL of 90% acetone were added to the pellet for a first cycle of lysis (50 Hz, 1 min). The extract was centrifuged (14,500×*g*, 5 min) and the supernatant collected in a new tube conserved on ice. Lysis cycles were then repeated with the addition of 500 µL of 90% acetone until the pellet became white. An absorbance spectrum between 400-800 nm was acquired from each final extract (UV 1900i double-beam UV-Vis Spectrophotometer, Shimadzu, Noisiel, France) and the chlorophyll *a* and chlorophyll c (c_1_+c_2_) concentrations were calculated according to Jeffrey and Humphrey (1975). All chemicals were of analytical grade and obtained from Sigma-Aldrich (Saint Quentin Fallavier, France).

### 2.5 Bright field microscopy for the determination of cell volumes

Images of *P. tricornutum* populations were acquired with a Zeiss Cell Discoverer 7 inverted microscope (Carl Zeiss France, Rueil-Malmaison, France). Cells from each sample were deposited in a well of a 24 well glass-bottom microplate and covered by a glass coverslip. Images were acquired with a ×50 water-immersion objective and a tube lens magnification of 0.5. Two mosaics of 5×5 images were collected for each sample.

Image analysis was automated with a homemade macro due to the large number of cells in the images. ImageJ plugin “Trainable Weka Segmentation” (Arganda-Carreras et al., 2017) was used to separate cells from the background in each image by machine learning algorithms. For the training phase, an image was randomly selected from the dataset. Around 20% of cells present in the image were registered manually in a “Cells” category, while an equal number of areas outside the cells were included in a distinct “Background, debris and bubbles” category. Training was performed using 3 filters, a Sobel filter for edge detection, a Maximum filter for texture detection and a Gabor filter detecting both edge and texture. The obtained classifier was used to create a probability map from each image, displaying the probability for each pixel to belong to a cell or to the background. The probability map was used as an input for the “Analyse particle” tool of ImageJ with a measurement of “Area”, “Shape descriptors”, “Fit ellipse” and “Feret’s diameter”, and a filtering by area (25 to 200 µm²) and by circularity (0 to 0.4). A further screening using shape descriptors “AR (Aspect Ratio)”, “Roundness” and “Solidity” (respective parameter values “**≥**3”, “**≤**0.3”, “**≥**0.55” for Pt1 and “**≥**3.5”, “**≤**0.3”, “**≥**0.5” for Pt4) was applied to eliminate false positives. Finally, the width of each cell was retrieved by taking the minor axis of an ellipse fitted to each cell area, and the length was retrieved as the Feret diameter of the cell shape.

Volume was then estimated using a prolate spheroid model (Hillebrand et al., 1999) with equation 2.


(Eq. 2) $$\text{Estimated volume}=\;\frac{\text{π}}{6}\;\times \;{\text{Width}}^{2}\times \text{Length}$$

where width and length are in µm, and estimated volume is in µm^3^.

### 2.6 Statistical analysis and graphical rendering

Comparison of the means within an ecotype was carried out using a one-way ANOVA test with the sampling time defined as the varying factor with 6 modalities (“D0”, “D3”, “D7”, “D11”, “h24” and “h72”) after controlling the normality of the data with a Shapiro-Wilks test. A pairwise post-hoc Fisher’s LSD test was used to define the levels of each modality as compared to the others and denote them with letters (a > b > c > d). Comparisons of the means between ecotypes were conducted using a *t*-test. In all cases, a *p*-value< 0.05 was retained as a criterion for statistical significance. For *t*-tests, magnitude of the *p*-value is rendered on the figures with one (*, 0.01< *p*-value< 0.05), two (**, 0.001< *p*-value< 0.01) or three (***, *p*-value< 0.001) asterisks.

The R package “ggplot2” (https://ggplot2.tidyverse.org/) was used for tracing violin plots and density maps. For box and whiskers plots, the delimitations were those of “ggplot2”: the line inside the box is the median, the ends of the box are the first and third quartile while the ends of the whiskers are the lowest (respectively highest) value between maximum (minimum) and Q3 + 1.5 × [interquartile range] (Q1 - 1.5 × [interquartile range]).

## 3 Results

The experimental design implemented for this study allows following the differential response of *P. tricornutum* Pt1 and Pt4 cells to a nitrogen (N) starvation (11 days) followed by a N resupply (72 hours).

### 3.1 Effects of varying nitrogen availability on the growth of *Phaeodactylum tricornutum* Pt1 and Pt4

**Figure 1:**
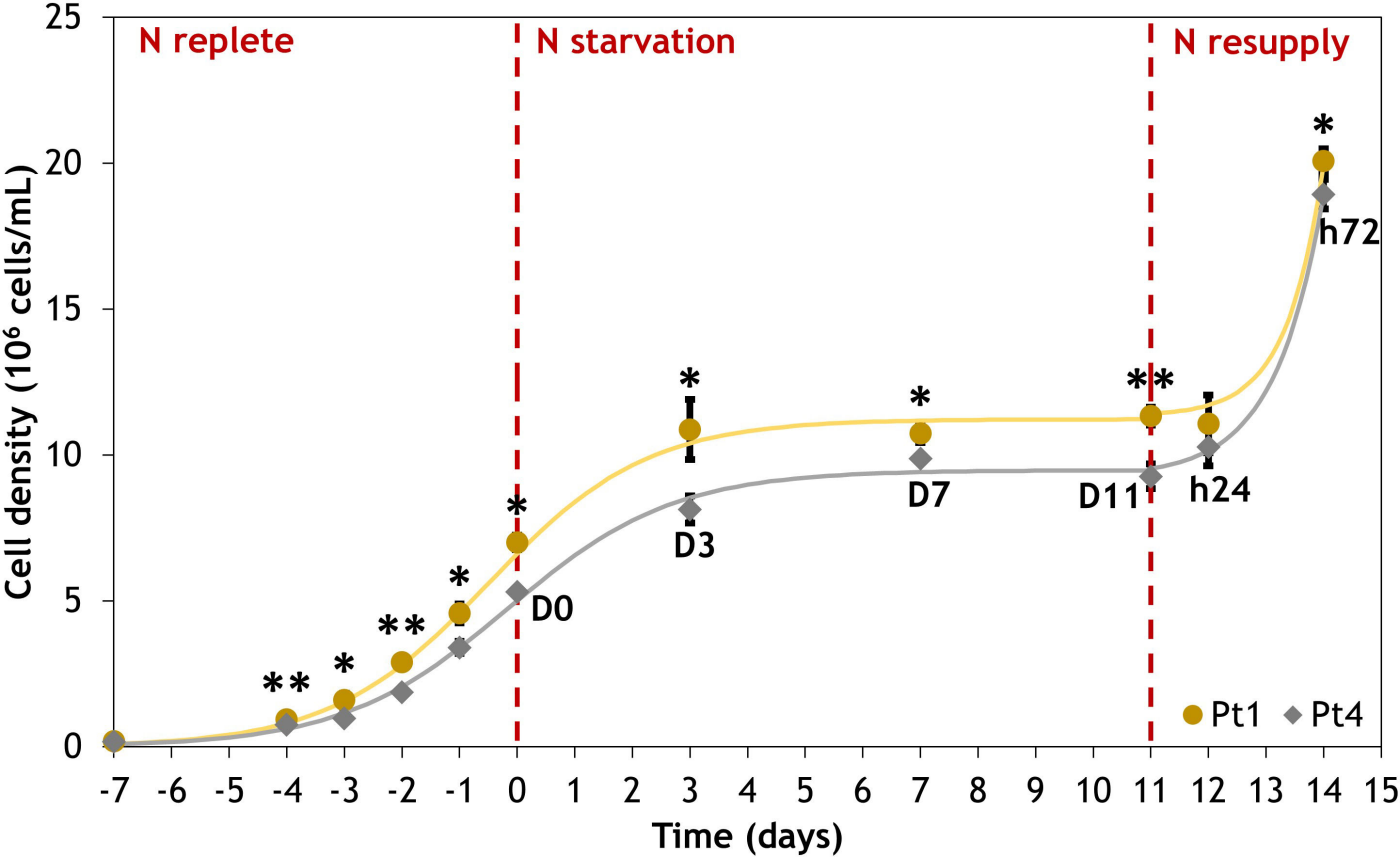
Evolution of cell density for Phaeodactylum tricornutum ecotypes Pt1 (yellow) and Pt4 (grey) under N replete, N starvation and N resupply conditions. Asterisks denote a difference between the two ecotypes (t-test: *0.01< p-value< 0.05; **0.001< p-value< 0.01). Each point is a Mean ± Standard deviation (n = 3).

The cell growth dynamics of the cultures are similar between ecotypes (**Figure 1**). The N replete conditions preculture permit exponential growth with division rates of 0.73 day^-1^ and 0.70 day^-1^ for Pt1 and Pt4, respectively. Cell division was halted by the switch to N_0_ medium that allowed only one doubling of cell density between D0 and D3, followed by a transient stand-by phase until D11 when N resupply resumed cell division, achieving approximately one doubling of cell density within 72 hours.

### 3.2 Evolution of intracellular organization during nitrogen starvation and resupply

**Figure 2:**
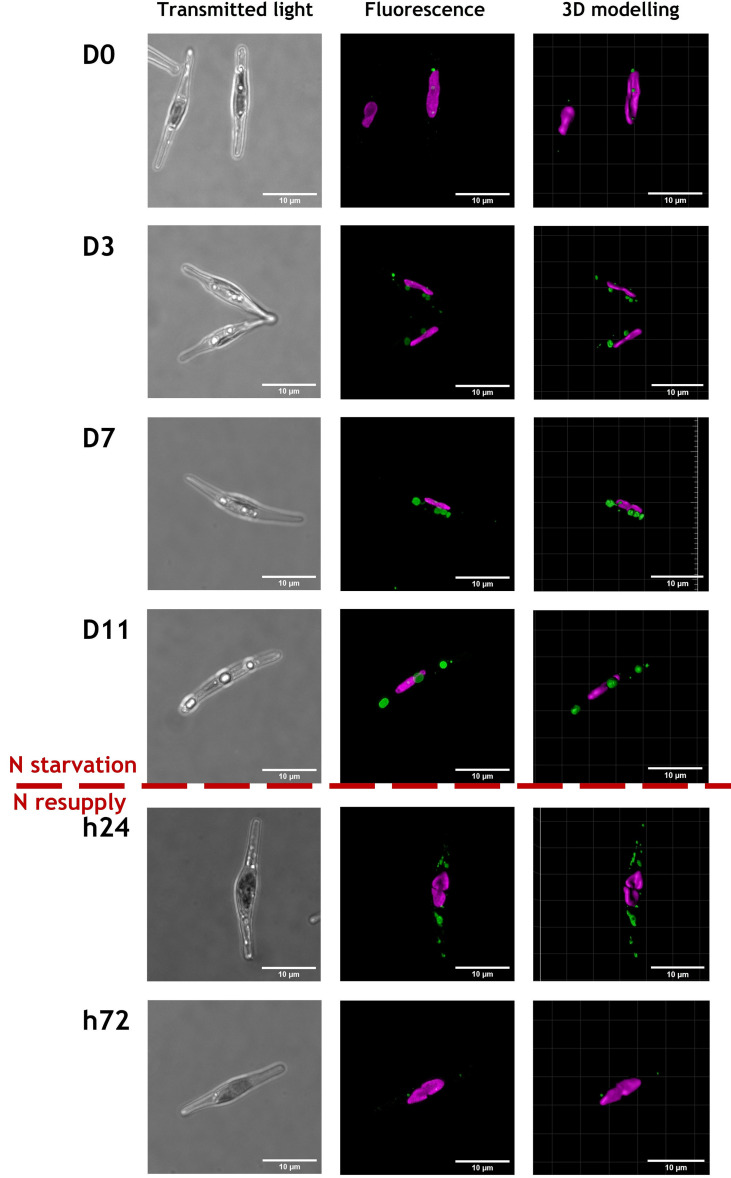
Representative images of cells from Pt1 ecotype during a nitrogen (N) starvation of 11 days followed by a N resupply of 3 days. Chlorophyll autofluorescence (650-700 nm) and Bodipy 505-515 fluorescence (505-550 nm) are displayed in magenta and green, respectively. Cells were selected from an overall number of 28, 25, 25, 24, 30 and 37 cells at respective timepoints from D0 to h72. The scale bar measures 10 µm.

**Figure 3:**
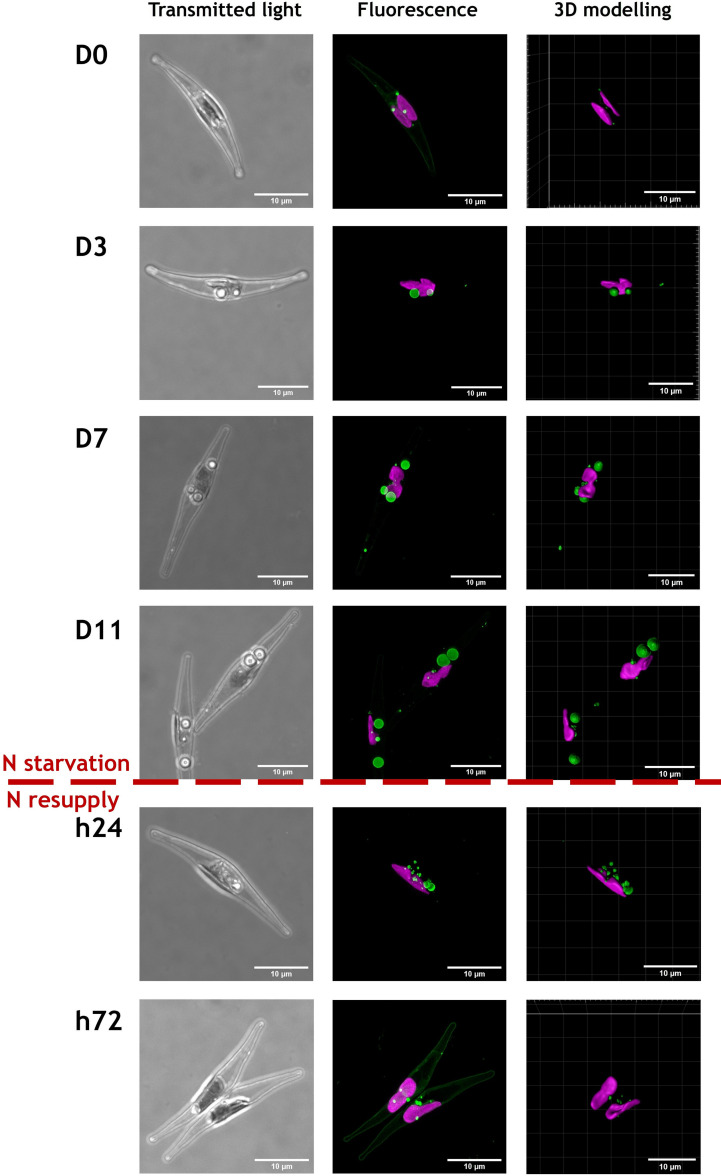
Representative images of cells from Pt4 ecotype during a nitrogen (N) starvation of 11 days followed by a N resupply of 3 days. Chlorophyll autofluorescence (650-700 nm) and Bodipy 505-515 fluorescence (505-550 nm) are displayed in magenta and green, respectively. Cells were selected from an overall number of 26, 24, 27, 26, 28 and 29 cells at respective timepoints from D0 to h72. The scale bar measures 10 µm.

CLSM observations were performed at the different time points of N starvation and N resupply phases to follow the cell general shape and chloroplast and LD locations in both ecotypes, based on chlorophyll and BODIPY 505/515 fluorescence (**Figures 2**, **3** and **S1**). Representative cells from 24-37 cells observed per time point, are displayed in **Figures 2**, **3**.

Regardless of the N supply, the chlorophyll autofluorescence image suggests the presence of a single elongated plastid in each cell meaning that the photosynthetic apparatus was maintained, at least partially (**Figures 2**, **3** and **S1**).

The most spectacular observed modification is the LDs dynamics with variation in both number and size of LDs. At D0, cells from both ecotypes contain a lower number of smaller LDs by comparison to those at D3 to D11. With regards to the N resupply, cells contained a large number of small LDs only 24 hours after the N resupply, which could indicate a fragmentation of the large LDs accumulated during starvation. At 72 hours after the N resupply, cells appeared to have returned to a similar state than at D0.

### 3.3 Nitrogen starvation induces an increase of lipid droplet volume along with a reduction in thylakoids while nitrogen resupply promotes a fast metabolic reversal

**Figure 4:**
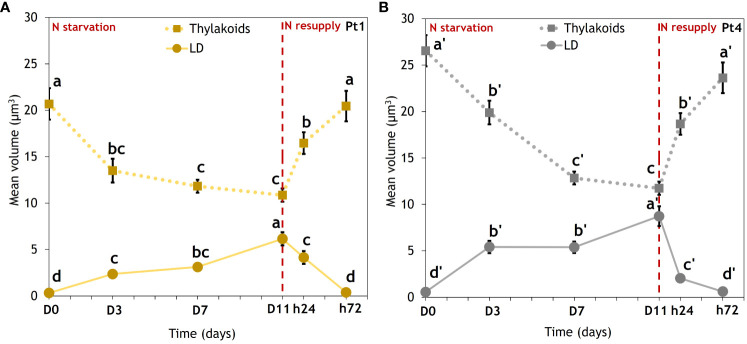
Evolution of thylakoids mean volume (dotted lines) and mean total volume of lipid droplets (solid lines) in cells of **(A)** Pt1 and **(B)** Pt4. Volumes were measured on 28, 25, 25, 24, 30 and 37 cells for Pt1 and 26, 24, 27, 26, 28 and 29 cells for Pt4 at respective timepoints from D0 to h72. Letters show differences between means at each time of sampling (ANOVA, p-value<0.05, LSD post-hoc test a>b>c>d (Pt1) and a’>b’>c’>d’ (Pt4)). Each value is a Mean ± Standard error (n = 24-37).

CLSM data allowed us to trace the evolution of the thylakoid and LD volumes per cell along the N starvation and N resupply experiment (**Figure 4**).

In both ecotypes, the volumes of these two subcellular compartments clearly followed opposite trends. During N starvation, thylakoids volume decreased while LD total volume increased. Under N resupply, cells experienced a very fast response, with thylakoids and LD volumes returning to an N replete (D0) value within 72 hours. When comparing ecotypes, Pt4 had a higher initial thylakoids volume. The Pt4 ecotype also reached a higher LD volume per cell than Pt1.

**Figure 5:**
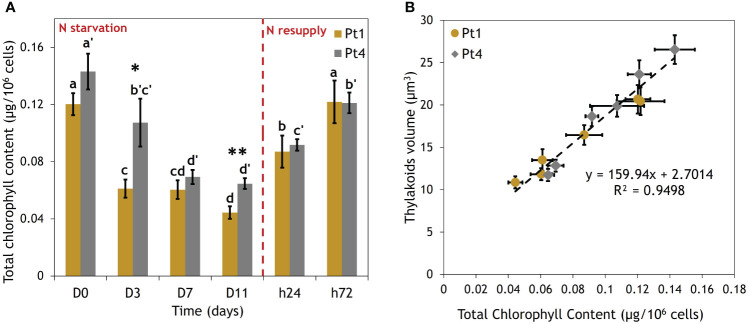
Correlation between thylakoids volume and total chlorophyll content per cell of Pt1 and Pt4 measured during a nitrogen (N) starvation followed by a N resupply. **(A)** Evolution of chlorophyll content, **(B)** Correlation between thylakoids volume and total chlorophyll content. Thylakoid were measured on 28, 25, 25, 24, 30 and 37 cells for Pt1 and 26, 24, 27, 26, 28 and 29 cells for Pt4 at respective timepoints from D0 to h72. Asterisks denote a difference between ecotypes (t-test: *0.01< p-value< 0.05; **0.001< p-value< 0.01), letters show differences between means at each time of sampling [ANOVA, p-value<0.05, LSD post-hoc test a>b>c>d (Pt1) and a’>b’>c’>d’ (Pt4)]. **(A)** Each value is a Mean ± Standard deviation (n = 3). **(B)** Thylakoid volume values are from **Figure 4**.

The effects of the N starvation and resupply on the thylakoids were further assessed by a total chlorophyll content assay (**Figure 5A**). Indeed, thylakoids volume and chlorophyll content were found to be linearly correlated (**Figure 5B**).

### 3.4 Ecotypes display highly different cell volumes that vary upon nitrogen starvation and resupply

One parameter that could explain the observed differences in lipid accumulation is the difference in cell volume between the two ecotypes. Under CLSM, we observed that in either N starvation or N resupply sampled Pt4 cells were longer than those of Pt1 with a length of ~30µm and ~20 µm, respectively (**Figures 2**. **3**). To go further, cell dimensions, i.e., length and width, were measured using bright field microscopy by sampling 2500-8000 cells per timepoint. Images were then processed automatically to retrieve dimensions of each cell and to estimate their volume using a prolate spheroid approximation of cell shape (Hillebrand et al., 1999).

**Figure 6:**
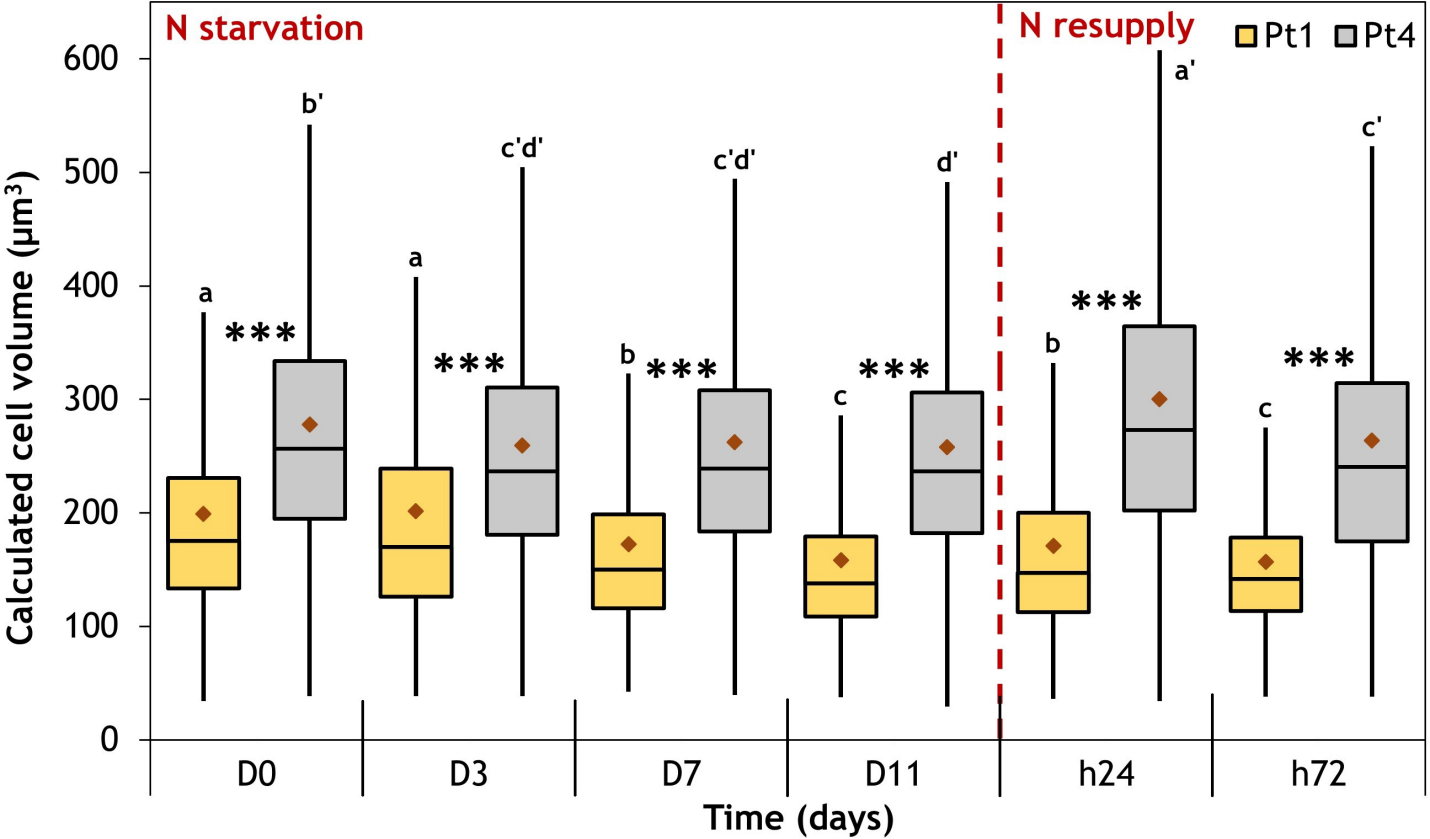
Cell volume variation in the populations of Pt1 (yellow) and Pt4 (grey) ecotypes measured during a nitrogen (N) starvation followed by a N resupply. Plots are traced from the cell volumes calculated for 4161, 4999, 6609, 7035, 7887 and 4919 cells of Pt1 and 2517, 4820, 5997, 5406, 6867 and 3807 cells of Pt4 at respective timepoints from D0 to h72. Asterisks denote a difference between ecotypes (t-test: *0.01< p-value< 0.05; **0.001< p-value< 0.01; ***p-value< 0.001), letters show differences between means at each time of sampling [ANOVA, p-value<0.05, LSD post-hoc test a>b>c (Pt1) and a’>b’>c’>d’ (Pt4)]. Box and whiskers plot: values plotted as given by R package ggplot2, the red diamond inside the plot is the mean.

The evolution of length, width, and volume of Pt1 and Pt4 cells along the 6 sampling points of the experiment are presented in **Table S1**. The two ecotypes clearly presented distinct ranges of variations, with width in the range 3.5-4 µm for Pt1 and 4-4.4 µm for Pt4, and length in the range 22.6-23.5 µm for Pt1 and 27.7-28.6 µm for Pt4. Hence, as observed by CLSM (**Figures 2**, **3**), Pt4 cells are overall larger than those of Pt1 (**Table S1**). It must be noted that each population contain cells of highly variable size (**Table S1**, **Figure 6**).

Along the experiment, mean cell volumes were not constant (**Table S1**; **Figure 6**, see significance letters). During N starvation, the mean volume decreases significantly for both ecotypes, going from 199 to 158 µm^3^ and from 278 to 258 µm^3^ between D0 and D11 for Pt1 and Pt4, respectively. While Pt4 mean cell volume immediately decreased to reach a “stand-by” minimum from D3 until D11, Pt1 cell volume decreased more progressively (**Figure 6**). At 24 hours after N resupply, cells of both strains experienced a significant transient increase in volume, reaching 171 µm^3^ and 300 µm^3^ for Pt1 and Pt4 respectively. At 72 hours after N resupply, the mean volumes of both types of cells decrease again. By tracing volume as a function of width and length, the variation in volume appeared to be driven by both width (R² = 0.98) and length (R² = 0.78) in Pt1 while only width (R² = 0.94) was correlated to volume in Pt4 (**Figure S2**).

### 3.5 Pt1 and Pt4 cells accumulate lipid droplets on distinct modes but degrade them similarly

**Figure 7:**
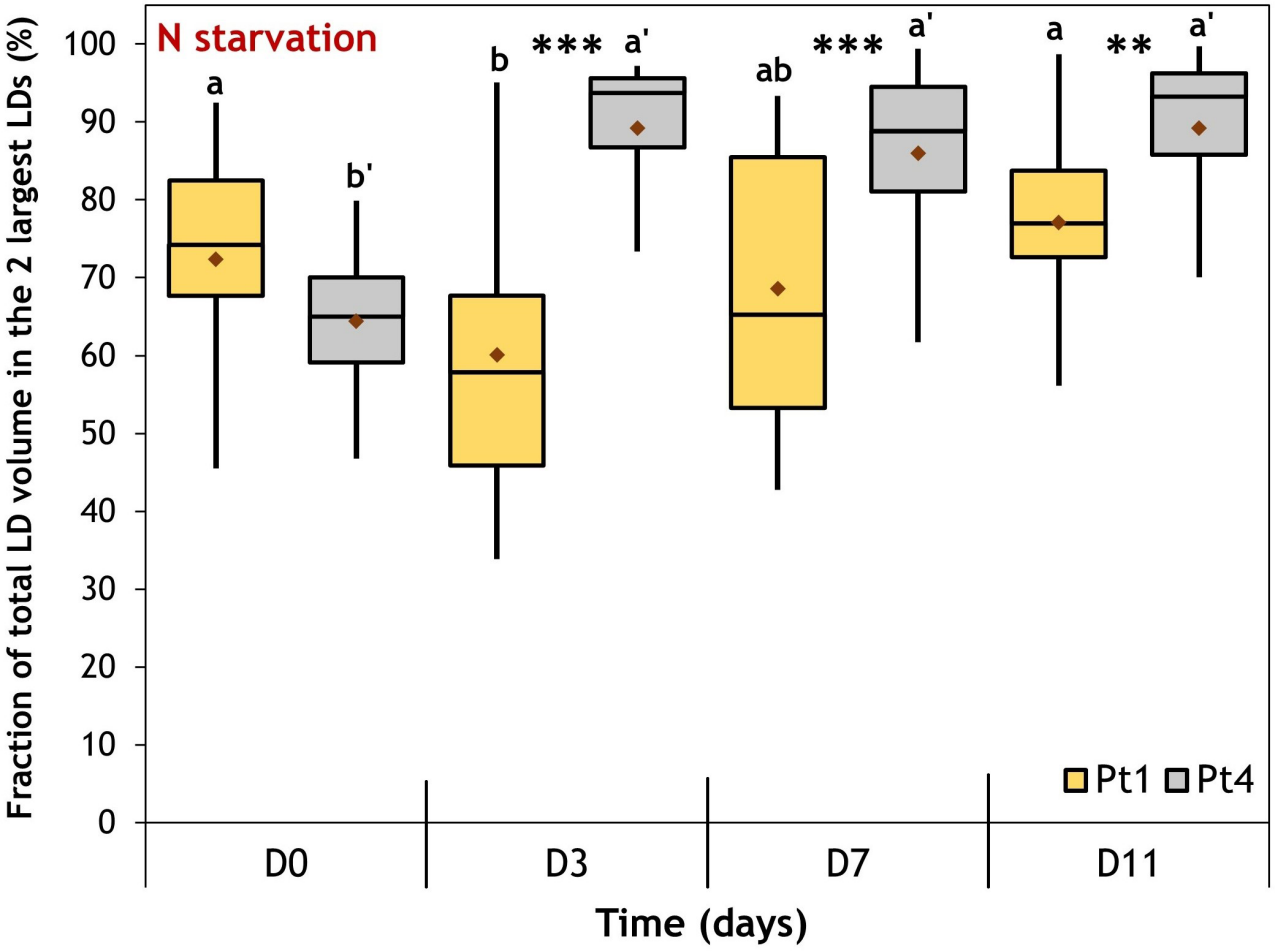
Evolution of the fraction of the total lipid volume per cell occupied by the 2 largest LDs during a nitrogen (N) starvation in Pt1 (yellow) and Pt4 (grey) ecotypes. Plots are traced from the fractions calculated for cells containing at least 3 LDs i.e. 18, 24, 24 and 23 cells of Pt1 and 12, 18, 23 and 23 cells of Pt4 at respective timepoints from D0 to D11. Asterisks denote a difference between ecotypes (t-test: *0.01< p-value< 0.05; **0.001< p-value< 0.01; ***p-value< 0.001), letters show differences between means at each time of sampling [ANOVA, p-value<0.05, LSD post-hoc test a>b (Pt1) and a’>b’ (Pt4)]. Box and whiskers plot: values plotted as given by R package ggplot2, the red diamond inside the plot is the mean.

CLSM data also provided insights into how lipids are distributed between LDs inside each cell. Indeed, it was previously described that Pt4 cells accumulate their lipids in two large LDs while Pt1 cells would form a higher number of smaller LDs (Leyland et al., 2020b; Scarsini et al., 2021). To assess these accumulation patterns, we defined the proportion of total LD volume comprised in the two largest LDs as an indicator for characterizing the inequality of lipid distribution between LDs in a cell. This indicator would tend to 100% when the majority of lipids is concentrated in only two LDs (very inequalitarian) while a lower value would indicate a repartition between a higher number of LDs (**Figure 7**).

At D0, the value of the inequality indicator suggests that the total volume of lipid droplets was shared between more than two LDs for both ecotypes. However, during N starvation, i.e when lipid accumulated, Pt4 cells concentrated most of their neutral lipids in the two largest LDs (median at ~90%), while Pt1 cells displayed a more equalitarian distribution between LDs (median at 60-75%).

**Figure 8:**
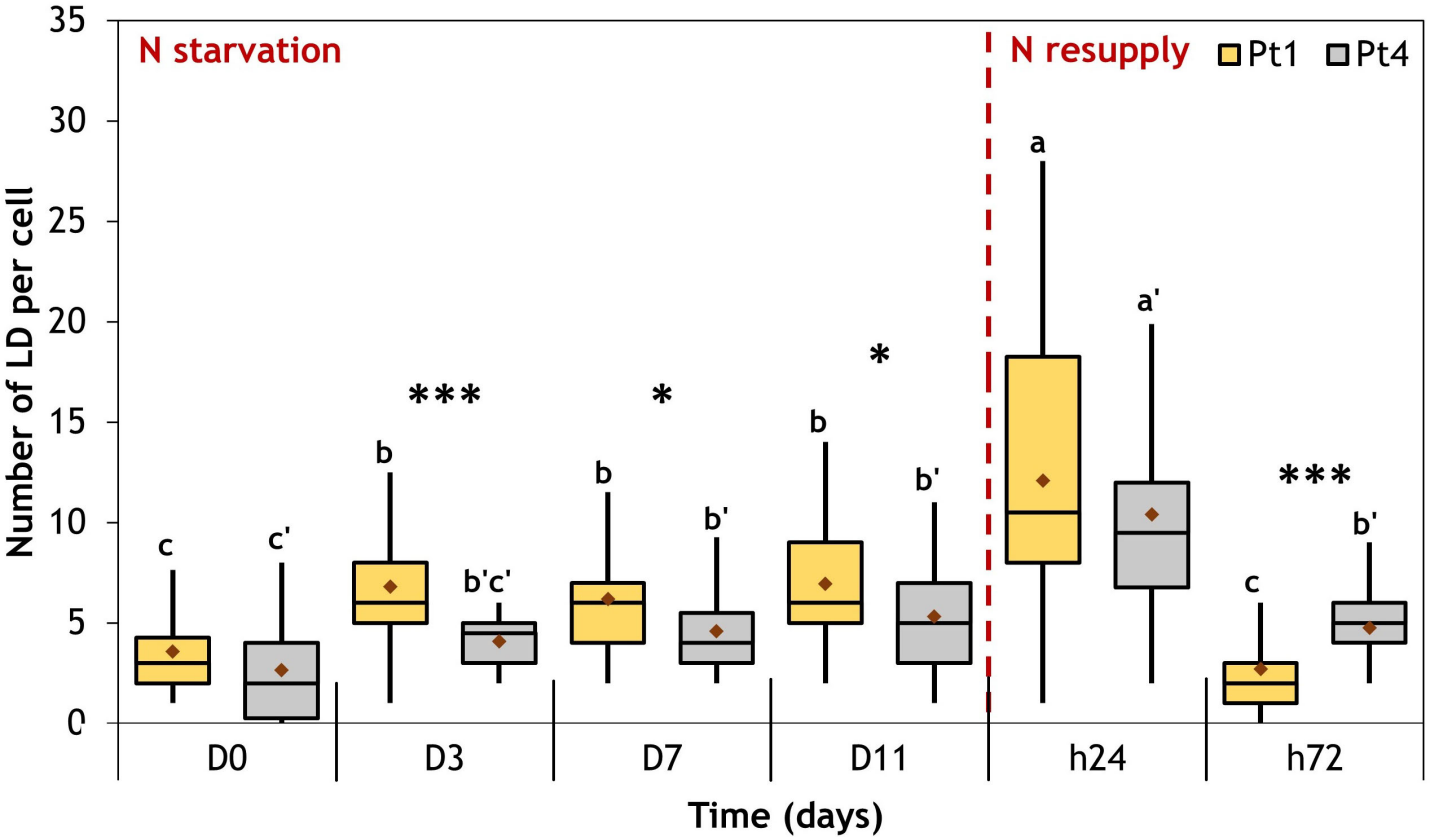
Evolution of the number of LD per cell during a nitrogen (N) starvation followed by a resupply in Pt1 (yellow) and Pt4 (grey) ecotypes. Plots are traced from the numbers of LDs collected in 28, 25, 25, 24, 30 and 37 cells for Pt1 and 26, 24, 27, 26, 28 and 29 cells for Pt4 at respective timepoints from D0 to h72. Asterisks denote a difference between ecotypes (t-test: *0.01<p-value<0.05; **0.001<p-value<0.01; ***p-value<0.001), letters show differences between means at each time of sampling [ANOVA, p-value<0.05, LSD post-hoc test a>b>c (Pt1) and a’>b’>c’ (Pt4)]. Box and whiskers plot: values plotted as given by R package ggplot2, the red diamond inside the plot is the mean.

As observed in **Figures 2**, **3**, the N resupply induced an increase in LD cellular quota and a decrease in LD individual size as compared to N starvation, suggesting a possible fragmentation of the large LDs formed during N starvation. To further investigate this phenomenon, the number of LDs per cell was followed during the whole experiment (**Figure 8**).

The two ecotypes followed similar patterns. At the beginning of N starvation (between D0 and D3), the number of LDs increased because of the formation of additional LDs (**Figures 2**, **3**, **8**). The LD cell quota value remained stable along lipid accumulation suggesting that the LDs are progressively filled with the *de novo* produced lipids. At 24 hours after starting N resupply, the LDs cell quota increased transiently, returning to the same situation as that of D0 at t72 for Pt1, while for Pt4, a slightly higher quota is observed at t72 as compared to D0 (**Figure 8**). The increase of the number of LDs per cell at h24 occurred while the total cellular LD volume decreased, suggesting that the numerous small LDs formed during h24 arose from the fragmentation of the few large LDs that were existing at the end of the N starvation period. This scenario is indeed supported by the variations of the individual mean LD volume (**Table 1**).

**Table 1:** *Variation of individual mean LD volume for ecotypes Pt1 and Pt4 under N starvation and resupply*.

Ecotype	Parameter	N replete	N starvation			N resupply	
		D0	D3	D7	D11	h24	h72
**Pt1**	LD count (LD/cell)	3.57 ± 2.08	6.80 ± 2.78	6.20 ± 2.65	6.96 ± 3.06	12.10 ± 7.62	2.70 ± 2.71
	V_Lipid (µm^3^/cell)	0.32 ± 0.35	2.36 ± 1.95	3.12 ± 1.95	6.15 ± 3.52	4.15 ± 3.80	0.39 ± 0.83
	V_Lipid/LD (µm^3^/LD)	**0.09 ± 0.04**	**0.35 ± 0.24**	**0.50 ± 0.35**	**0.88 ± 0.60**	**0.34 ± 0.25**	**0.14 ± 0.24**
**Pt4**	LD count (LD/cell)	2.65 ± 2.43	4.08 ± 1.25	4.59 ± 2.15	5.31 ± 2.62	10.39 ± 6.15	4.76 ± 1.57
	V_Lipid (µm^3^/cell)	0.56 ± 0.33	5.41 ± 3.28	5.39 ± 3.20	8.72 ± 5.42	2.03 ± 1.43	0.61 ± 0.32
	V_Lipid/LD (µm^3^/LD)	**0.21 ± 0.10**	**1.32 ± 0.65**	**1.17 ± 0.57**	**1.64 ± 0.77**	**0.20 ± 0.16**	**0.13 ± 0.06**

Results are expressed as Mean ± Standard deviation (n = 24-37). Significance of the variation in LD count and V_lipid are reported in **Figure 8**, **4**. respectively.

### 3.6 The distribution of lipid droplet volumes reveals the presence of two dynamic populations

**Figure 9:**
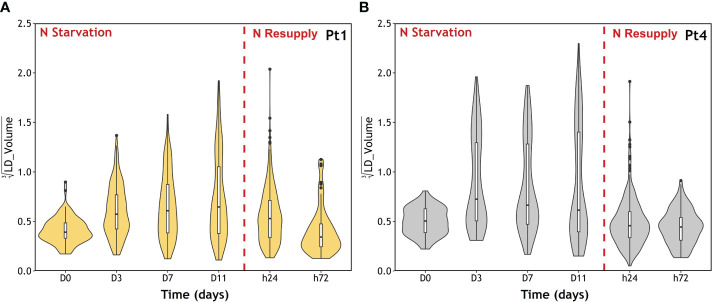
LD population volume distribution during the course of a nitrogen (N) starvation followed by a N resupply in Pt1 **(A)** and Pt4 **(B)** ecotypes. Plots are traced from the volumes collected in 100, 170, 155, 167, 389 and 79 LDs for Pt1 and 69, 96, 124, 138, 291 and 138 LDs for Pt4 at respective timepoints from D0 to h72. The violin plot outline represents the density of observations as a function of volume. Volumes are displayed as cubic roots to allow a better visualization. Box and whiskers plot: default parameters in R package ggplot2.

As the LD cellular quota changed over the timepoints of the experiment (**Figure 8**), and that the repartition of volumes between LDs during the accumulation varies along the experiment for both ecotypes (**Figure 7**), the distribution of individual LD volumes along the N starvation and N resupply periods was traced to investigate patterns of LD accumulation (**Figure 9**).

Both ecotypes displayed an initial population of small LDs at D0. Then, during N starvation, a second population of large LDs appeared, progressively in Pt1 and immediately in Pt4. Finally, after the N resupply, the second population of LDs in both ecotypes almost completely vanished at h24 and totally at h72, reaching a distribution similar to that of D0.

### 3.7 The distance between lipid droplets and the plastid indicates a movement of lipid droplets inside the cell upon nitrogen resupply

**Figure 10:**
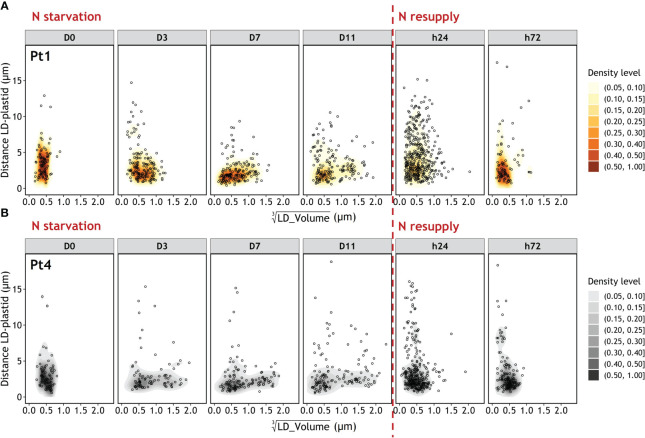
Evolution of the Euclidian distance between LD and plastid as a function of LD volume during a nitrogen (N) starvation followed by a N resupply in Pt1 **(A)** and Pt4 **(B)** ecotypes. Density levels represent the probability of finding a data point within the corresponding area. Maps are traced from the volumes and coordinates collected in 100, 170, 155, 167, 389 and 79 LDs for Pt1 and 69, 96, 124, 138, 291 and 138 LDs for Pt4 at respective timepoints from D0 to h72. Volumes are displayed as cubic roots to allow a better visualization.

As degradation mechanisms may involve the movement of LDs inside the cell, the distances between LD and plastid were plotted as a function of LD volumes (**Figure 10**). The initial population of small LDs is located close (≤5 µm) to the plastid. Similarly, as the large LDs appear, they are also mostly found close to the plastid. In contrast, 24 hours after the N resupply, a vast number of small LDs are observed to be distant (>5 µm) from the plastid. At h72 the LD-plastid distance as a function of LD volume is similar to that of D0.

## 4 Discussion

In this study, we analyzed the formation and degradation dynamics of LDs in two genetically distant ecotypes of *P. tricornutum*, namely Pt1 and Pt4 (Rastogi et al., 2020). The evolution of intracellular organization, especially LD number and size appears to be variable between the two ecotypes. We also investigated the relationship between cell volume and the extent of LD accumulation. Finally, we provided insights into the so far elusive LD degradation pathways.

### 4.1 Ecotypes Pt1 and Pt4 comparison is a useful tool to observe differences in qualitatively similar strains

Several strains of *P. tricornutum* are able to drastically change their shape under conditions of varying temperature or salinity, shifting between the main triradiate, fusiform and oval morphotypes (Ovide et al., 2018; Song et al., 2020; Galas et al., 2021). In the culture conditions used in this study, the major morphotype observed for both Pt1 and Pt4 is the fusiform one. As already described for fusiform cells (Flori et al., 2017; Broddrick et al., 2019), they both contain a unique plastid located at the center of the cell and displaying a bilobed shape (**Figures 2**, **3** and **S1**). Both strains also share the same major biochemical compounds including chlorophyll *a* and *c*, fucoxanthin as a major carotenoid, the β1,3 glucan called chrysolaminarin as a storage carbohydrate and C16:0, C16:1 n-7 and C20:5 n-3 as major fatty acids in their total lipids fatty acid profiles (Abida et al., 2015; Popko et al., 2016; Huang et al., 2019; Scarsini et al., 2021).

Regarding their differences, a genome comparison highlighted that Pt4 was genetically diverging from all the 9 other investigated Pt ecotypes (Rastogi et al., 2020). We observed that the strains had similar growth rates in the tested conditions (**Figure 1**). However, by the time of the transition between N replete and N starvation phases, Pt1 cultures reached a higher cell density (7 x 10^6^ cells/mL) than Pt4 cultures (5.3 x 10^6^ cells/mL). In parallel, even including a high variability within the populations, we observed that the mean volume of Pt4 cells was 140% of that of Pt1 cells (D0, **Figure 6**). For a given nutrient quantity available in the medium, Pt4 cultures would hence reach a lower cell density than Pt1 cultures at the stationary phase as more nutrients are incorporated per cell. Such a pattern of cell density was observed in a previous comparison of Pt1 and Pt4 ecotypes (Scarsini et al., 2021). The same study also observed that Pt4 cultures had a higher N intake rate per cell, possibly due to an altered mode of nitrogen acquisition permitted by a higher number of copies of a nitrate assimilation gene (Phatr3_EG02286, nitrite reductase) as highlighted by Rastogi et al. (2020). In line with these results, lower cell densities were found in Pt4 compared to Pt1 but identical dry biomass accumulation was observed under N starvation, resulting in a higher content of dry mass per cell (**Figure S3**).

Considering cell dimensions, we observed that both cell length and width were higher in Pt4 cells as compared to Pt1 cells (**Table S1**). The divergence could have arisen from an initial difference in the ecotypes. Indeed, the two ecotypes were initially isolated from different environments, namely, the ocean for Pt1 and brackish water in rock pools for Pt4 (Martino et al., 2007).

In addition, according to the photosynthetic characteristics observed through the PI curves obtained from both ecotypes it appeared that, even though the features (composition and size) of the light-harvesting antenna were similar as shown by the similar initial slope of the curves, the values of maximum photosynthesis and photoacclimation parameter showed that Pt1 reached photosynthesis saturation at a higher irradiance as compared to Pt4 (**Supplementary Data S1**). When light intensity was over the E_k_ value, Pt1 was not photoinhibited while Pt4 was, as demonstrated by the diminution of the electron transfer rate values. Therefore, the difference observed in photosynthetic characteristics further highlights the divergence between strains.

It is interesting to note that the length and the width of cells appear to be inconsistent between studies. For example, in the first global comparison of the 10 main ecotypes of *P. tricornutum*, de Martino et al. (2007) reported that Pt1 and Pt4 fusiform morphotypes do not differ in length (range 19-24 µm) and that Pt1 cells had a higher width (2.9-3.9 µm) than Pt4 cells (2.5-3.6 µm). By contrast, a more recent report showed that Pt4 cells had the same length, but a higher width than Pt1 ones, resulting in a higher volume (Scarsini et al., 2021). These differences could have arisen from phenotypic plasticity as a result of an acclimation to each laboratory culture conditions such as culture medium, photoperiod, light irradiance or temperature.

The drifting of strains maintained in different laboratories along replications on the long term could also be a variation factor. A varying acclimation and, hypothetically, the accumulation of mutations would lead to strains with different physiological characteristics. Indeed, we also traced photosynthesis-irradiance curves of two different strains of Pt1, our Pt1 and another Pt1 strain (Pt1_Wuhan). As can be seen in **Supplementary Data S1**, Pt1_Wuhan displays intermediate characteristics between those of Pt4 and those of Pt1. It was also previously shown that these two Pt1 strains diverged in their lipid accumulation strategy (Scarsini et al., 2021).

Finally, another possible variation factor for cell dimensions is the measurement technique employed. Using a high-resolution electron microscopy approach associated to 3D reconstruction, a recent study reported a volume for Pt1 cells of 32.6 ± 2.4 µm^3^ which correspond to the lower end of our observations (**Figure 6**) (Uwizeye et al., 2021). Whether such a “low” value is due to sampling bias, to different culture conditions, to the precision of the method or to inherent characteristics of this clone of Pt1 remains to be determined. Nonetheless, in our study, the sampling bias was reduced by using an automated approach to measure all the cells present in our bright field microscopy images.

### 4.2 How does cell volume influence the lipid accumulation dynamics under nitrogen starvation?

Exposing microalgae to N starvation is a common way for inducing neutral lipid accumulation and storage in LDs. In this condition, either ecotype reacted similarly by decreasing their thylakoids volume in parallel to increasing their total LD volume (**Figure 4**). The thylakoid and LD volumes can be considered as an estimator of total chlorophyll (Broddrick et al., 2019) and neutral lipid (Jaussaud et al., 2020) contents, respectively. N starved cells shift their metabolism from an active to a stand-by mode to wait for more favorable conditions. In the transition phase between N replete and N starved conditions, cells increase their N assimilation capacity and in the meantime recycle N containing molecules including chlorophylls (Scarsini et al., 2022). The decrease in chlorophyll content, also permits the reduction of their photosynthetic machinery to a minimal point, to prevent the accumulation of reactive oxygen species. In parallel, cells reorient their carbon central metabolism towards the production of neutral lipids, in part to store electrons produced by the residual photosynthesis and in part by recycling the fatty acid of plastidial membrane lipids produced during the dismantling of thylakoid membranes (Abida et al., 2015; Remmers et al., 2017; Huang et al., 2019; Scarsini et al., 2022).

The onset of cell response to N starvation is also observed by an arrest of growth after one doubling of cell density between D0 and D3 (**Figure 1**). This division is permitted by the use of intracellular N storage (Shoman, 2015). During the N starvation phase, cell volumes were shown to decrease in both ecotypes (**Figure 6**). A possible hypothesis for this decrease would be that cell size is related to cell division. Under a division regime, mother cells would enlarge before dividing into two daughters. This would be coherent with the fact that both Pt1 and Pt4 volume variations were well-correlated with width variations (**Figures S2**) as, during division, *P. tricornutum* daughter cells are formed side by side, separating along a longitudinal dividing plane (Tanaka et al., 2015). However, as Pt1 volume is also correlated to its length (**Figure S2A**), this explanation is not sufficient at least for this ecotype. The reduction in cell volume under nitrogen stress is in line with what was observed in Pt4 in a previous study (Huang et al., 2019), but the same study showed that an increase in cell volume was observed under phosphorus limitation, even though this condition also limits growth. Additionally, other types of environmental change were shown to influence both cell size and growth rate of diatoms. Some conditions produced similar effects to nitrogen deficiency. A reduction in irradiance induced a reduction in cell size and growth rate in *P. tricornutum* and 6 other diatoms (Thompson et al., 1991) and an iron deficiency reduced cell volume and growth rates in *Pseudo-nitzschia* spp. (Marchetti and Harrison, 2007). Others had phosphorus limitation-like effects such as an increase in temperature that induced an increase in cell size but a decrease in growth rate in *Thalassiosira pseudonana* (Sheehan et al., 2020), or a decrease in salinity that provoked the same effects in *Corethron histrix* (Aizdaicher and Markina, 2010). The multiple factors that influence cell volume and their relative contribution should hence be determined, as this parameter directly influence available intracellular space for the plastid but also for LD accumulation.

Indeed, previous experiments highlighted that cell volume of *P. tricornutum* could be a limiting factor of neutral lipid accumulation by limiting the size of the largest LDs (Jaussaud et al., 2020): as the large LDs reach a maximal volume determined by cytosolic space left available after considering the volumes of plastid, mitochondria and nucleus, further lipid accumulation occurs through smaller LDs that occupy the remaining gaps (Jaussaud et al., 2020). However, this report did not consider neither the variation in the relative size of cellular organelles, the relative size of the vacuole, nor the variations in cell volume. More recently, a 3D reconstruction showed that under replete conditions, around 45% of total cytosolic volume is occupied by the plastid, nucleus and mitochondrion leaving at most 55% for LDs (Uwizeye et al., 2021). Under N starvation, we observed that the reduction in chlorophyll content is highly correlated to a reduction in the volume of thylakoids (**Figure 5**). This reduction in thylakoid volume could be concomitant with a reduction in global plastid volume, as this organelle was previously shown to shrink under N starvation in the diatom *Cyclotella meneghiniana* (Rosen and Lowe, 1984). Our data showed that the reduction in thylakoid volume had a higher amplitude than that of the increase in total LD volume: between D0 and D11, the thylakoid volume loss is of 9.8 and 14.8 µm^3^, while the LD volume gain is of 5.8 and 8.2 µm^3^ for Pt1 and Pt4, respectively (**Figure 4**).

Although the reduction in volume of cells from both ecotypes under N starvation must also be considered (mean reduction of 41 µm^3^ for Pt1 and 20 µm^3^ for Pt4; **Figure 6**), the proportion of total cell volume occupied by LDs remains low, going from 0.16 and 0.20% at D0 to 3.9 and 3.4% at D11 for Pt1 and Pt4, respectively (calculated from **Figures 4**, **6**).

To summarize, Pt4 cells were larger than those of Pt1 and they also had a higher thylakoid volume at D0 as well as a higher LD volume at D11. In addition to a limitation by available intracellular space, we hence suggest a new hypothesis for explaining a higher lipid accumulation in large cells: these cells have a more voluminous plastid that could provide more thylakoid membrane lipids to be recycled into LDs during N starvation. This hypothesis could be tested by comparing a wider range of ecotypes with varying thylakoid under N replete conditions volume and their respective total LD volume accumulation.

The two hypotheses are not mutually exclusive as the neutral lipids that are stored in LDs are likely synthesized from both recycling of preexisting lipids and *de novo* synthesis. Indeed, some reports highlighted a major role in lipid recycling (Remmers et al., 2018; Scarsini et al., 2022) as genes related to lipid synthesis are mostly downregulated under N limited/starved conditions and genes related to lipid recycling are upregulated. Other studies show that the extant of lipid accumulation must include *de novo* synthesis as a major mechanism as the reduction in polar lipids observed under N starvation is not high enough to account for the increase in neutral lipids (Abida et al., 2015; Haslam et al., 2020). However the source of carbon for *de novo* synthesis must be already present in the cell, for example the backbone of recycled amino acids or storage carbohydrates, as carbon fixation by photosynthesis is almost abolished under N starvation, as shown by a near-stability of C intracellular quota (Scarsini et al., 2022) and a decrease in photosynthetic parameters along with the downregulation at both transcript and protein levels of most of the genes involved in photosynthesis (Longworth et al., 2016; Remmers et al., 2018; Scarsini et al., 2022). Therefore, in depth biochemical investigation should be conducted to determine the respective importance of the two (or more) limiting factors to the extent of LD accumulation.

### 4.3 The distinct lipid droplet accumulation strategies of ecotypes Pt1 and Pt4

The CLSM data presented in this contribution highlight similarities and differences between the strategies of LD formation in the two ecotypes, Pt1 and Pt4. Both strains had a basal population of small LDs at D0 (**Figure 9**). Upon N starvation, the cells from both strains produced additional LDs, but with two different strategies highlighted by the repartition of lipids between LDs (**Figure 7** and **Table 1**) and the distribution of LD volumes (**Figure 9**). Until now, it was described that Pt1 cells accumulated multiple small LDs that appear by waves and stop increasing in volume after 3 to 4 days, while Pt4 cells produced only 2 large ones which volume increased during the N starvation (Wong and Franz, 2013; Jaussaud et al., 2020; Leyland et al., 2020b). Our high-resolution approach coupled to 3D modelling confirmed and refined these observations. We described that Pt4 concentrates its lipids in two large LDs accounting for 90% of total LD volume (**Figure 7**), creating an additional LD subpopulation to the basal population containing the remaining 10%. Considering LD distribution, the observations on Pt1 are also in line with previous work with the presence of a higher number of smaller LDs (Jaussaud et al., 2020). The progressive growth of the population of large LDs in both strains from D3 to D11 of N starvation (**Figure 9**) is coherent with Pt4 previous observations (Wong and Franz, 2013; Leyland et al., 2020b) but not with those in Pt1 (Jaussaud et al., 2020). Such a discrepancy for Pt1 could have arisen from a variation in culture conditions and/or strain variations between laboratories. Overall, the main difference between Pt1 and Pt4 lipid accumulation strategies resides in how the population of larger LDs behaves.

### 4.4 After nitrogen resupply, both ecotypes consume their lipid droplets using a similar fragmentation mechanism

While N starvation induced a stand-by state in Pt1 and Pt4 cells, N resupply triggered a fast recovery in both ecotypes, with a decrease in LD volume in parallel of an increase in thylakoid volume (and total chlorophyll content) and with growth resuming between time points h24 and h72 after N resupply (**Figure 1**, **4**, **5A**). This behavior is in line with what was observed in the few resupply experiments previously conducted on *P. tricornutum* Pt1 and Pt4 (Valenzuela et al., 2013; Jallet et al., 2020; Scarsini et al., 2022; Taparia et al., 2022), where N resupply induced an immediate increase in pigments, and a simultaneous (Pt1, Jallet et al., 2020; Pt4, Scarsini et al., 2022) or delayed to 48h (Pt1; Valenzuela et al., 2013) decrease in neutral lipids. In all experiments, growth resumed within around 48 hours as we also observed.

In all resupply experiments, cell division follows the consumption of neutral lipids. However, the mechanisms underlying this phenomenon are still elusive. The fragmentation of LDs that we proposed to explain the results observed 24 hours after N resupply (**Table 1**) has not been described previously in microalgae or diatoms. Actually, the nutrient resupply experiments conducted on the diatom *Fistulifera solaris* JPCC DA0580 showed a rather different behavior, with the two large LDs of this species progressively shrinking without signs of fragmentation (Nomaguchi et al., 2018; Nonoyama et al., 2019). To our knowledge, such a fragmentation phenotype of large LDs under lipid consuming conditions has only been described in murine 3T3-L1 adipocytes (Paar et al., 2012; Chitraju et al., 2017). In *P. tricornutum*, fragmentation could be a way to enhance the surface to volume ratio and obtain a more efficient access of lipases to the neutral lipids contained in LDs.

Consumption of lipids could occur by lipolysis (direct consumption of lipids at the surface of LDs), lipophagy (autophagy-based bulk consumption in vacuoles), or a combination of both. As an evidence of lipolysis, it was previously shown that the inactivation of non-vacuolar lipases in *P. tricornutum* led to an accumulation of neutral lipids (Barka et al., 2016; Li et al., 2018). Recently, evidence towards the importance of lipophagy have also been obtained. One major LD protein of *P. tricornutum*, the Stramenopile lipid droplet protein (StLDP), contains a domain of interaction with ATG8, a crucial protein for autophagy (Leyland et al., 2020a). Indeed, in *Nannochloropsis oceanica*, another Stramenopile microalga model, the major lipid droplet protein (NoLDSP) was shown to interact with ATG8 under lipid consumption conditions. The authors also observed patterns of lipophagy using transmission electron microscopy describing the engulfment of LDs in vacuoles (Zienkiewicz et al., 2020). As a further clue for the role of StLDP in lipophagy, two recent studies reported that mutant lines of *P. tricornutum* Pt1 (Taparia et al., 2022) and Pt4 (Yoneda et al., 2023) whose StLDP gene was knock-out by CRISPR-Cas9 showed an impaired LD degradation upon nutrient resupply. Our data highlighted that a high number of LDs were located far from the plastid at timepoint h24 (**Figure 10**). This movement of LDs away from the plastid could indicate an association with the vacuoles, as in *P. tricornutum* fusiform cells vacuoles expand towards the arms (Liu et al., 2016; Schreiber et al., 2017), and could suggest lipophagy. However, LDs could also associate to peroxisomes or the mitochondria as a way to direct the fatty acids produced by lipolysis to the β-oxidation pathway, although in *P. tricornutum* the mitochondria is tightly associated with the plastid (Flori et al., 2017; Uwizeye et al., 2021). Indeed, lipolysis and lipophagy are not mutually exclusive and signs of both of them should be further investigated through electron microscopy. Finally, an inhibitor assay conducted in the diatoms *F. solaris* and *P. tricornutum* Pt1 (Nonoyama et al., 2019) showed that the extant of lipid consumption was reduced but not suppressed after nutrient resupply when autophagy was inhibited, which is a further proof that lipolysis and lipophagy act in combination.

## 5 Conclusions

In this work, a combination of microscopicmethods were used to follow and compare cells from ecotypes Pt1 and Pt4 experiencing a N starvation and resupply conditions. Nitrogen starvation produced a standby mode where an accumulation of LDs occurs at the expense of the plastid. In this context, the two ecotypes adopted distinct strategies regarding the repartition of accumulated lipids between LDs. Additionally, the extant of lipid accumulation was possibly linked to cell volume as larger cells contain a larger plastid to be disassembled as a source for neutral lipid production. Finally, when resupplied with nitrogen, fast recovery was achieved in both ecotypes through a similar fragmentation process that could be linked to lipophagy. Our findings lead to several further questions: (1) what are the underlying determinants of the maximal size of LDs in both ecotypes? (2) which molecular mechanism governs the fragmentation of LDs? (3) what is the respective importance of lipolysis and lipophagy in LD degradation? Future investigation shall tackle these aspects by comparing the response of the ecotypes to a silencing of specific LD proteins or proteins involved in lipid degradation.

## Data availability statement

The original contributions presented in the study are included in the article/**Supplementary Material**, further inquiries can be directed to the corresponding author.

## Author contributions

VM: Conceptualization, Data curation, Formal analysis, Investigation, Methodology, Visualization, Writing- original draft, Writing- review & editing. JH: Conceptualization, Data curation, Formal analysis, Methodology, Supervision, Validation, Visualization, Writing- review & editing. MC: Investigation, Methodology, Writing- review & editing. SG: Investigation, Writing- review & editing. AL: Conceptualization, Data curation, Methodology, Software, Writing- review & editing. CC: Conceptualization, Data curation, Methodology, Software, Writing- review & editing. MBe: Conceptualization, Investigation, Methodology, Writing- review & editing. LG: Conceptualization, Methodology, Supervision, Validation, Writing- review & editing. BS: Methodology, Validation, Writing- review & editing. JM: Conceptualization, Data curation, Methodology, Supervision, Validation, Visualization, Writing- review & editing. MBa: Conceptualization, Supervision, Validation, Writing- review & editing. LU: Conceptualization, Data curation, Supervision, Validation, Visualization, Writing- review & editing.
