# Supplementary material for: Comparison of two Phaeodactylum tricornutum ecotypes under nitrogen starvation and resupply reveals distinct lipid accumulation strategies but a common degradation process
Source: Front Plant Sci. 2023 Sep 22;14:1257500. doi: 10.3389/fpls.2023.1257500 (PMC10556672; doi:10.3389/fpls.2023.1257500)
Supplement: Supplementary file 1 [file DataSheet_1.pdf]

Comparison of two *Phaeodactylum tricornutum* Ecotypes under Nitrogen Starvation and Resupply Reveals Distinct Lipid Accumulation Strategies but a Common Degradation Process

Victor Murison et al.

Supplementary Material

Supplementary Data S1

Supplementary Figures S1-S3

Supplementary Table S1

Supplementary Data S1

To determine how the different strains of *P. tricornutum* react to increasing light intensities, rapid light curves (PI curves) during which diatoms are quickly exposed to a range of light levels while relative electron transport rate (rETR) is estimated from the fluorescence intensity (White and Critchley, 1999). The measurement were performed using Dual-PAM 100, Walz, Germany) using the default routine. Figure SD1 presents the rapid light curves obtained with the different strains of *P. tricornutum* tested: the Pt1 and Pt4 ecotypes used in this study, and an additional Pt1 strain, Pt1_Wuhan kindly provided by Pr H. Hu (Key Laboratory of Algal Biology, Institute of Hydrobiology, Chinese Academy of Sciences, Wuhan China).

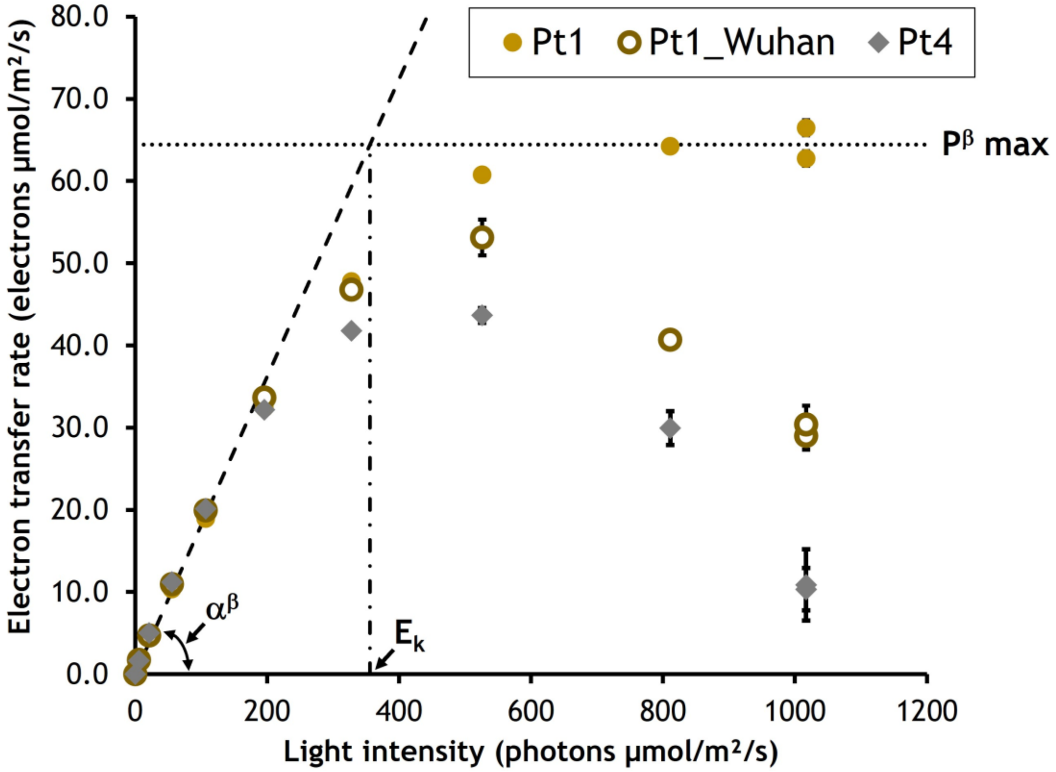

Figure SD1: Photosynthesis irradiance (PI) curves of the three strains Pt1, Pt1_Wuhan and Pt4.

Relative electron transfer rate was used as a proxy for photosynthetic rate and traced as a function of light intensity. The photophysiological parameters are displayed on the graph: α_β is the initial slope, P^βmax the maximum photosynthetic rate (approximated by the electron transfer rate) and E_k the light saturation parameter that indicates the onset of saturation. Mean of three biological replicates are presented.

Individual PI-curves were fitted according to the model proposed by (Eilers and Peeters, 1988). From these data, the following parameters were calculated:

- α_β *parameter* is defined as the initial slope of the rapid light curve. This parameter is usually considered as proportional to the efficiency in which microalgae harvest light (Eilers and Peeters, 1988; Nguyen-Deroche et al., 2012).
- P^{β}_{Max} *parameter* is defined as the asymptotic value of light-curve. It reflects the maximal rETR (Eilers and Peeters, 1988; Nguyen-Deroche et al., 2012).
- E_k *parameter* is defined as the irradiance corresponding to the intercept between α_β and P^{β}_{Max}. E_k is considered as an index for photosynthetic light saturation. Photon flux densities above the E_k value can cause photooxidative stress resulting in an increase of photoinhibition (Horton and Ruban, 1992; Barber, 1995; Niyogi, 1999).

Table SD1: Photophysiological parameters derived from the photosynthesis irradiance curves of the three strains Pt1, Pt1_Wuhan and Pt4.

Strain	P^{β} max (µmol electrons/m²/s)	α^{β} (µmol electrons/µmol photons)	E_k (µmol photons/m²)
Pt1	64.4	0.1809	356
Pt1 Wuhan	53.2	0.1896	281
Pt4	43.7	0.1924	227

The values of α_β of the different strains were very close (Table SD1), indicating that the composition and the size of the light-harvesting antenna complexes were not different among the strains because they were all growing at the same growth light intensity. In contrast, they differed in the values of P^{β}_{Max} and therefore in E_k (Table SD1). According to the measurements, the strain performing best photosynthesis at the optimum light intensity was Pt1 followed by Pt1-Wuhan, and Pt4. In these growth conditions, $E_{k,Pt4}$ was evaluated to be 227 µmol photons $m^{-2}s^{-1}$ (Table SD1), a value intermediates between those found for this ecotype grown at 30 or 300 µmol photons $m^{-2}s^{-1}$ (Heydarizadeh et al., 2017). When the light intensity was over the E_k values, only Pt1 was not photoinhibited and the two other strains failed to maintain the electron transfer rate at the maximum capacity as demonstrated by the diminution of the rETR values (Fig. SD1). This contrasts with its behaviour when grown under 300 µmol photons $m^{-2}s^{-1}$ (Heydarizadeh et al., 2017).

References:

Barber, J. (1995). Molecular basis of the vulnerability of photosystem II to damage by light. *Australian Journal of Plant Physiology* 22, 201–208.

Eilers, P. H. C., and Peeters, J. C. H. (1988). A model for the relationship between light intensity and the rate of photosynthesis in phytoplankton. *Ecological Modelling* 42, 199–215. doi: 10.1016/0304-3800(88)90057-9.

Heydarizadeh, P., Boureba, W., Zahedi, M., Huang, B., Moreau, B., Lukomska, E., et al. (2017). Response of CO_2 -starved diatom *Phaeodactylum tricornutum* to light intensity transition. *Phil. Trans. R. Soc. B* 372, 20160396. doi: 10.1098/rstb.2016.0396.

Horton, P., and Ruban, A. V. (1992). Regulation of photosystem II. *Photosynth Res* 34, 375–385. doi: 10.1007/BF00029812.

Nguyen-Deroche, T. L. N., Caruso, A., Le, T. T., Bui, T. V., Schoefs, B., Tremblin, G., et al. (2012). Zinc affects differently growth, photosynthesis, antioxidant enzyme activities and phytochelatin synthase expression of four marine diatoms. *The Scientific World Journal* 2012, 1–15. doi: 10.1100/2012/982957.

Niyogi, K. K. (1999). Photoprotection revisited: genetic and molecular approaches. *Annu. Rev. Plant. Physiol. Plant. Mol. Biol.* 50, 333–359. doi: 10.1146/annurev.arplant.50.1.333.

White, A. J., and Critchley, C. (1999). Rapid light curves: A new fluorescence method to assess the state of the photosynthetic apparatus. *Photosynth Res* 59, 63–72. doi: 10.1023/A:1006188004189.

Supplementary Figure S1

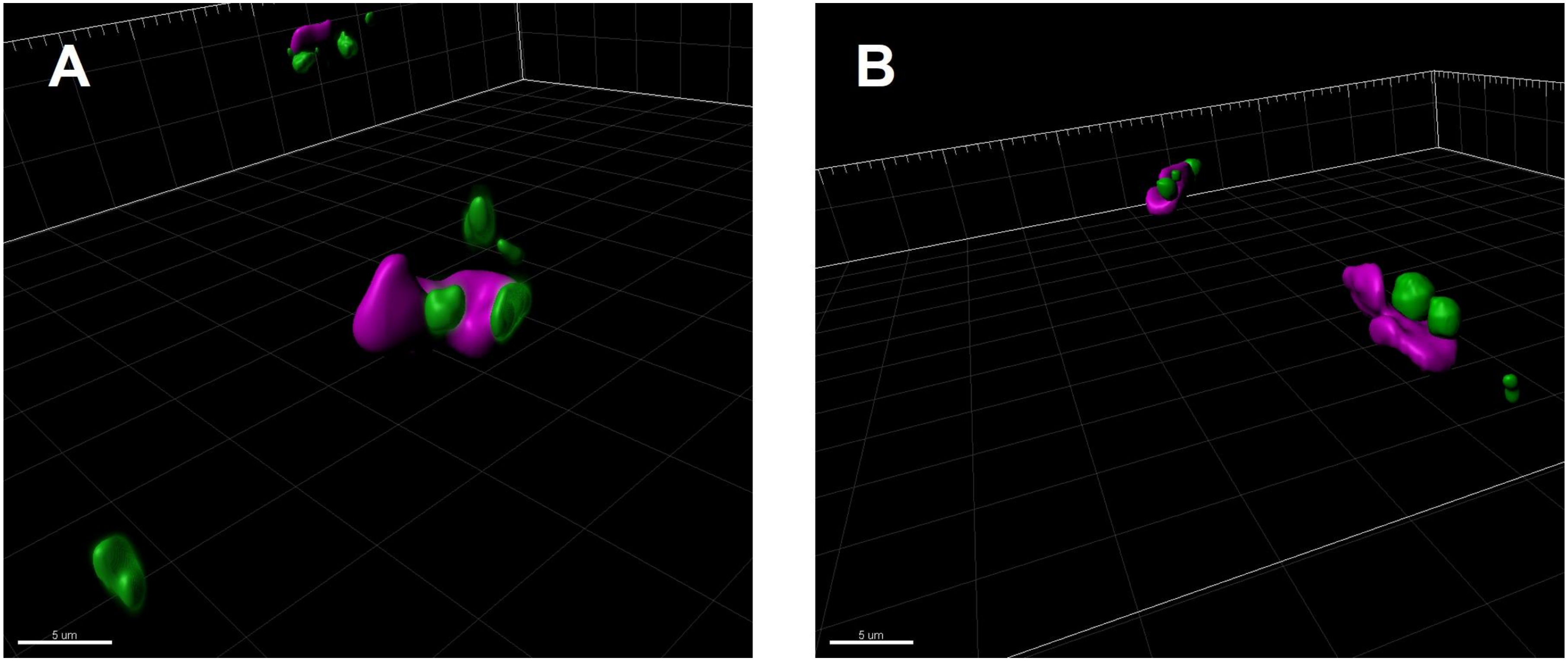

Examples of views from the 3D models of *P. tricornutum* (A) Pt1 (at D7) and (B) Pt4 (at D11) showing the bilobed shape of plastids. The plastid was modelled as a magenta 3D shape where chlorophyll autofluorescence was detected, lipid droplets are modelled as green 3D shapes where Bodipy 505-515 fluorescence was detected. The scale bar measures 5µm.

Supplementary Figure S2

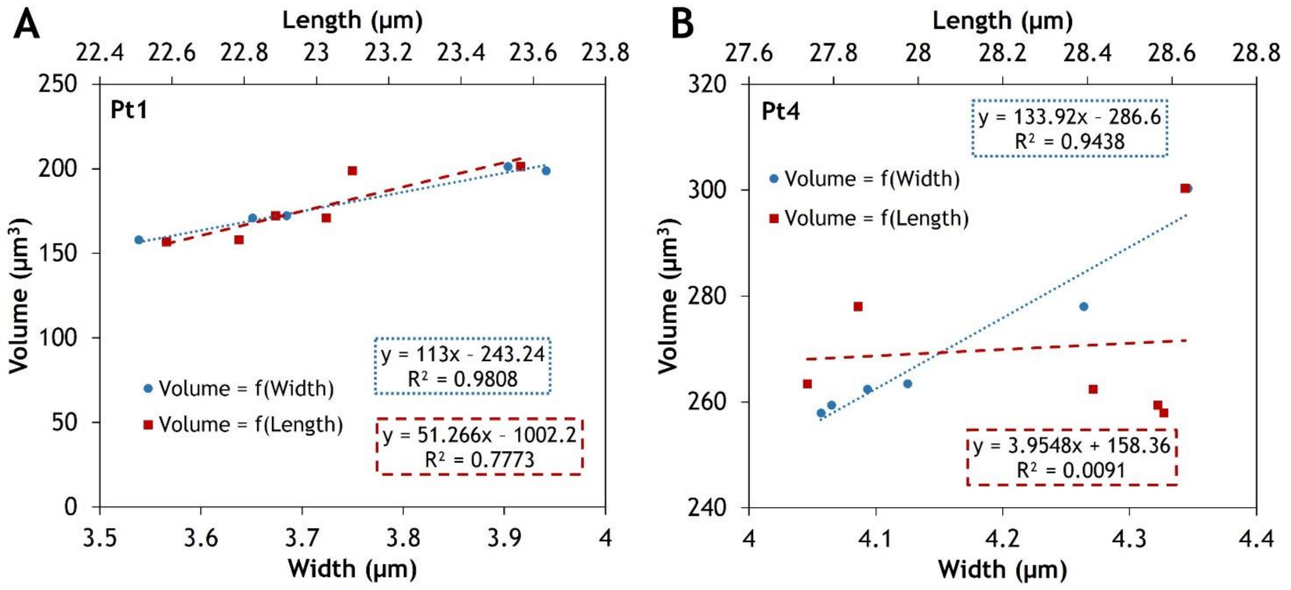

Relationship between cell volume and cell length and width in populations of (A) Pt1 and (B) Pt4 sampled at different timepoints of N starvation and N resupply. Each value is a Mean (n=2517-7887).

Supplementary Figure S3

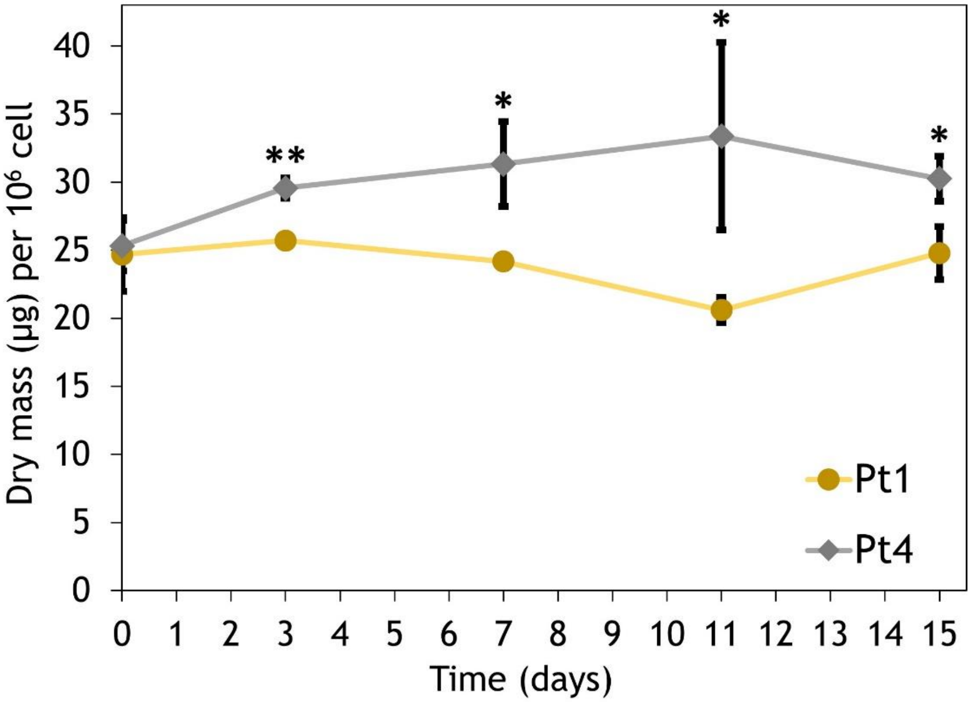

Evolution of dry weight content during a N-starvation experiment on cultures of Pt1 and Pt4 ecotypes. Cultures were conducted in biological triplicate under a continuous white LED-light illumination at 20°C. Dry weight was determined by harvesting 20 mL of culture by filtration on 0.22 µm GF-C glass fiber filters (Whatman). The filters were washed twice with ammonium formate (1 mol/L) to remove the salt and dried for 24h at 60°C. Asterisks denote a difference between the two ecotypes (*t-test*: *, 0.01<*p-value*<0.05; **, 0.001<*p-value*<0.01).

Supplementary Table S1

Evolution of width, length and estimated volume of Pt1 and Pt4 cells during a N starvation followed by a N resupply.

Time	Width (µm)		Length (µm)		Estimated volume (µm³)	
	Pt1	Pt4	Pt1	Pt4	Pt1	Pt4
D0	3.94 ± 0.79	4.26 ± 0.76	23.10 ± 3.51	27.86 ± 5.18	198.87 ± 104.87	278.04 ± 129.00
D3	3.90 ± 0.89	4.06 ± 0.77	23.56 ± 3.42	28.57 ± 5.02	201.40 ± 119.68	259.43 ± 124.52
D7	3.68 ± 0.73	4.09 ± 0.75	22.89 ± 3.57	28.41 ± 5.31	172.42 ± 96.46	262.41 ± 130.22
D11	3.54 ± 0.67	4.06 ± 0.72	22.79 ± 3.70	28.58 ± 5.04	158.03 ± 90.45	257.92 ± 120.54
h24	3.65 ± 0.77	4.35 ± 0.85	23.03 ± 3.49	28.63 ± 5.05	171.04 ± 99.39	300.36 ± 150.01
h72	3.57 ± 0.61	4.12 ± 0.85	22.58 ± 3.70	27.74 ± 5.06	156.88 ± 76.36	263.50 ± 140.50

Results are expressed as Mean ± Standard deviation (n = 2517-7887)
